# Diagnostic accuracy of pancreatic stone protein in patients with sepsis: a systematic review and meta-analysis

**DOI:** 10.1186/s12879-024-09347-4

**Published:** 2024-05-06

**Authors:** Biwei Mai, Lirong Zhou, Qi Wang, Bo Ding, Yifeng Zhan, Shanqing Qin, Naiyun Zhu, Zhuangxing Li, Zhixian Lei

**Affiliations:** Paediatric Intensive Care Unit, Hainan Women and Children’s Medical Center, Changbin Road, Xiuying District, Haikou, Hainan, Hainan 570100 China

**Keywords:** Pancreatic stone protein, Sepsis, Diagnosis, Sensitivity, Specificity

## Abstract

**Background:**

Sepsis is a common syndrome of multiorgan system dysfunction secondary to the dysregulated inflammatory response to infection. The role of pancreatic stone protein (PSP) in diagnosing sepsis has been investigated in previous studies. The meta-analysis aimed to comprehensively investigate the diagnostic value of PSP in identifying sepsis.

**Methods:**

PubMed, Web of Science, Embase, Cochrane Library, and China National Knowledge Infrastructure (CNKI), were systematically searched. Studies investigating the diagnostic performance of PSP were included. Pooled sensitivity, specificity, positive Likelihood Ratio (+ LR) and negative Likelihood Ratio (-LR), diagnostic odds ratio (DOR), and area under the curve (AUC) of summary receiver operating characteristic (SROC) were calculated.

**Results:**

The sensitivity of PSP was 0.88 (95% CI: 0.77–0.94), and the pooled specificity was 0.78 (95% CI: 0.65–0.87). Pooled + LR, -LR, and DOR were 4.1 (2.3, 7.3), 0.16 (0.07, 0.34), and 26 (7, 98). The AUC value for the SROC of PSP was 0.90 (0.87, 0.92). The pooled sensitivity, specificity, + LR and - LR, and DOR for PSP among neonates were 0.91 (95% CI: 0.84, 0.96), 0.66 (95% CI: 0.58, 0.74), 3.97 (95% CI: 0.53, 29.58), 0.13 (95% CI: 0.02, 1.00), and 31.27 (95% CI: 0.97, 1004.60).

**Conclusions:**

This study indicates that PSP demonstrated favorable diagnostic accuracy in detecting sepsis. Well-designed studies are warranted to ascertain the value of PSP measurement to guide early empirical antibiotic treatment, particularly in neonates.

**Supplementary Information:**

The online version contains supplementary material available at 10.1186/s12879-024-09347-4.

## Background

Sepsis is a clinical syndrome characterized by organ dysfunction due to dysregulated host response to infection; it is still one of the most common causes of mortality in critically ill patients [1–3]. The mortality rate of sepsis is estimated to be between 20% and 40%, and long-term complications are common, including kidney failure, liver failure, depression, and neurocognitive impairment [4–6]. It is estimated that there are over 19 million cases of sepsis and 5 million sepsis-related deaths each year, with the majority occurring in low and middle-income countries [7]. Early identification of sepsis is crucial for improving prognosis and preventing relevant complications [8].

Biochemical markers can help practitioners identify sepsis as early as possible. Nevertheless, commonly used biomarkers, including C-reactive protein (CRP) and procalcitonin, have been proven inaccurate in detecting sepsis in previous meta-analyses [9, 10]. CRP showed promising sensitivity in detecting infection, nevertheless, it is only specific if a high cutoff level is used, which it will in turn reduce sensitivity [11]. Procalcitonin (PCT) has been determined to have the highest performance as a biomarker for the diagnosis and prognosis of sepsis. However, it may be elevated in many diseases other than infection, especially after surgery and trauma [12, 13]. Better biomarkers are warranted to increase the value of sepsis detection. Pancreatic stone protein (PSP), a 144-amino-acid glycoprotein, might be a suitable biomarker for sepsis; however, the physiological role of PSP has not been elucidated yet [14–17]. PSP is mainly secreted by pancreatic acinar cells and also secreted by intestinal and gastric cell subsets [18]. A study illustrated that PSP is an inflammatory mediator that can bind and activate neutrophils, thereby acting as an acute phase protein in response to injury in the early stages of infection [16].

Several investigations have explored the diagnostic capabilities of PSP in detecting sepsis, demonstrating favorable outcomes [19–23]. However, these studies were limited concerning their sample sizes and the population involved. This meta-analysis aimed to draw a comprehensive knowledge of the diagnostic value of PSP in sepsis identification.

## Methods

### Search strategy and selection criteria

This meta-analysis was conducted in adherence to the Preferred Reporting Items for Systematic Reviews and Meta-analysis (PRISMA) [24]. From the inception date until April 20, 2023, a comprehensive search was executed across multiple electronic databases, encompassing PubMed, Web of Science, Embase, the Cochrane Library, and China National Knowledge Infrastructure (CNKI), considering literature in both English and Chinese languages. The search strategy incorporated an array of keywords and synonyms: pancreatic stone protein, PSP, sepsis, Bloodstream Infection, Bloodstream Pyemia, Pyemias, Pyohemia, Septicemia, Blood Poisoning, Blood Severe Sepsis. A flowchart for search, screening, and eligibility identification was constructed based on the PRISMA guidelines. Inclusion criteria were as follows: (1) subjects suspected of having sepsis; (2) clear documentation of a reference standard; (3) a 2 × 2 contingency table can be formed categorizing participants with true positive (TP), false positive (FP), false negative (FN), true negative (TN) results on PSP test. Exclusions pertained to case reports, reviews, editorials, conference abstracts, animal studies, or studies with unextractable data. The entire process of database querying and study selection was independently executed by two reviewers, with any disagreements resolved through iterative discussions until consensus was achieved.

### Data extraction and risk of bias assessment

The following study level data were retrieved: name of the first author, publication year, country, sample size, median or mean age, proportion of the female, standard reference, counts of participants with TP, FP, FN, and TN outcomes. The risk of bias assessment of studies was evaluated according to the Quality Assessment of Diagnostic Accuracy Studies-2 (QUADAS-2) [25]. Two authors independently extracted data from included studies and appraised the risk of bias of these studies; disagreement was solved through consultation with a third investigator.

### Statistical analysis

Statistical analyses at the study level were executed using Stata 14.0 software and Meta-DiSc 1.4. The following summary measures were computed, accompanied by their corresponding 95% confidence intervals (CIs): pooled sensitivity, specificity, positive Likelihood Ratio (+ LR), negative Likelihood Ratio (-LR), diagnostic odds ratio (DOR), and the area under the curve (AUC) derived from the summary receiver operating characteristic (SROC) curve. To gauge the heterogeneity among the included studies, Cochran’s Q statistic and the I2 index were employed. According to established thresholds, heterogeneity was categorized as insignificant (I^2^ = 0–25%), low (I^2^ = 25–50%), moderate (I^2^ = 50–75%), or high (I^2^ = 75–100%) [26]. Publication bias was visually inspected through the construction of funnel plots and further subjected to statistical assessment utilizing Deeks’ method [27]. Additionally, a sensitivity analysis was carried out to gauge the influence of individual studies on the aggregate results. Statistical significance was set at a *p*-value less than 0.05.

## Results

### Study selection and characteristics

**Fig. 1:**
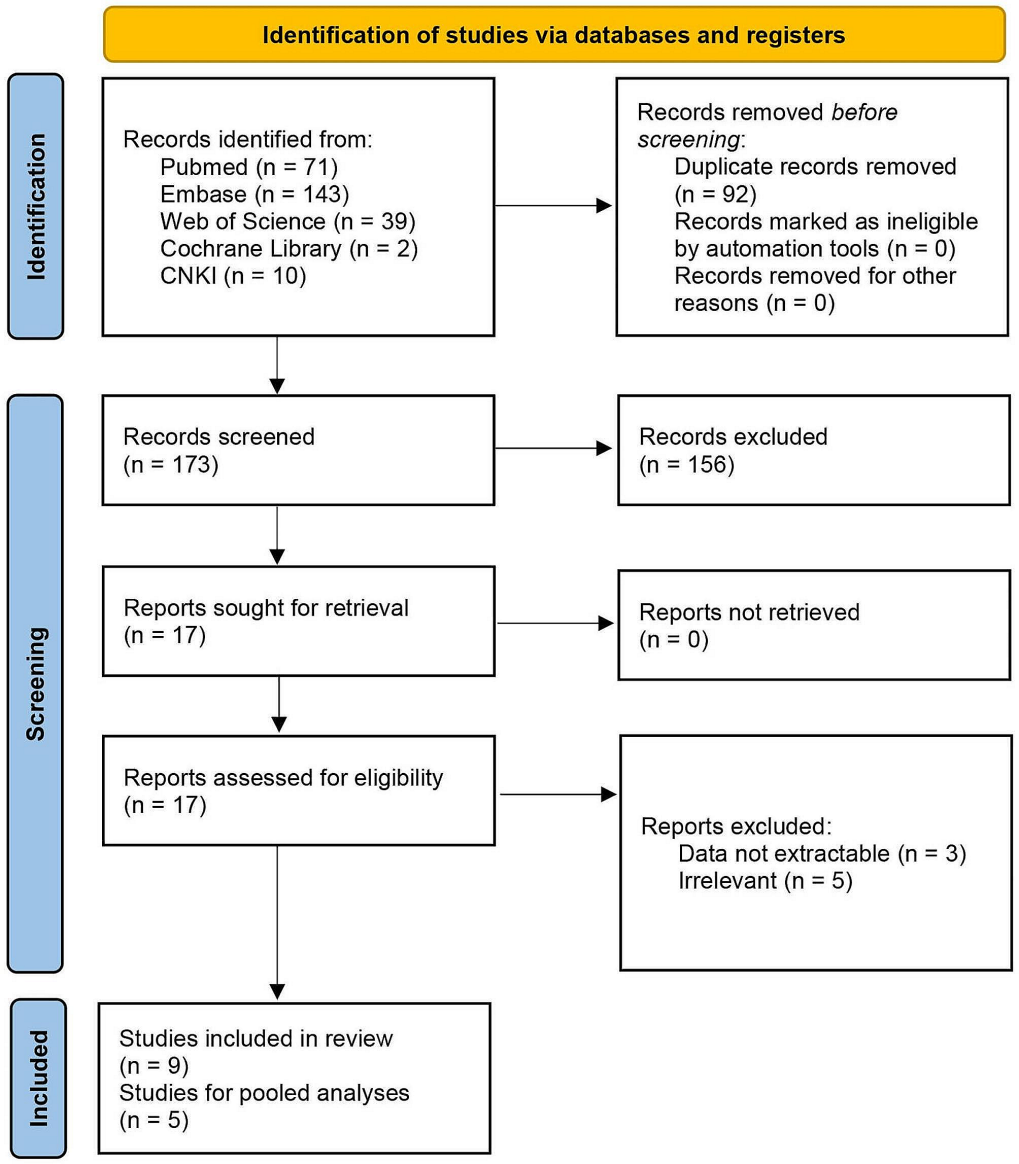
Flowchart of the literature search

**Fig. 2:**
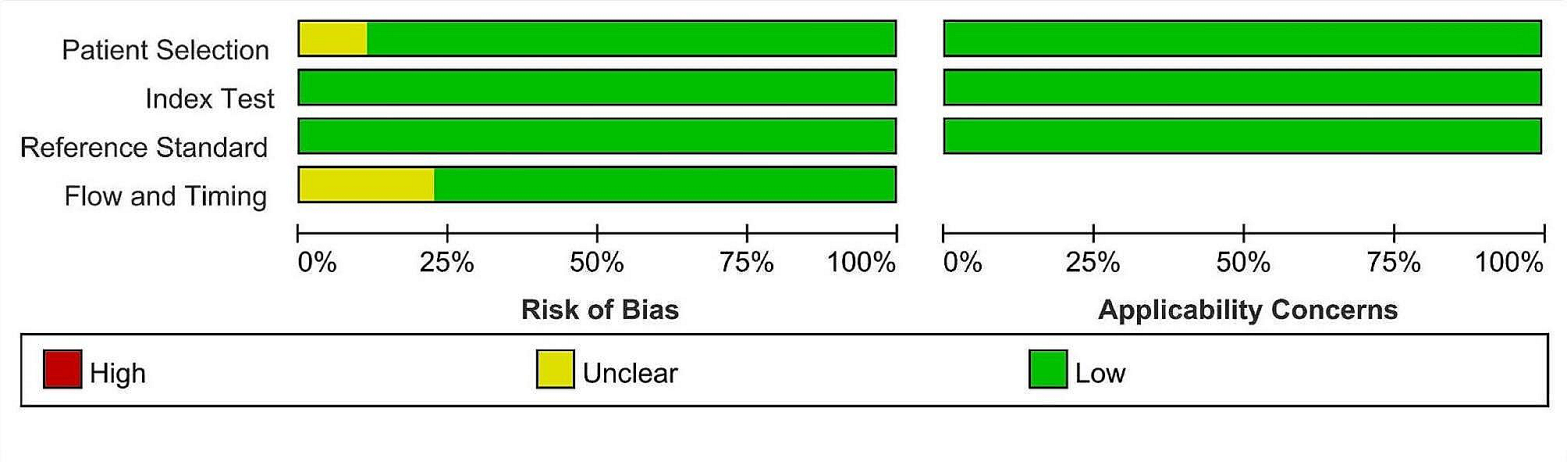
Summary of risk of bias in included studies

The literature search yielded 265 articles. Among them, 92 duplicated citations were removed, and another 156 studies were excluded through an initial screening of the title and abstract. The full-text reading of the remaining 17 articles identified nine studies with 1364 participants eligible for inclusion in this study [8, 11, 19–23, 28, 29] and five studies in meta-analysis. Figure 1 displays the flowchart of the database search and study selection. Six studies were performed among adults; the other 3 included studies were conducted among newborns. The coverage of countries included the UK, Switzerland, Netherlands, Spain, Egypt, France, and Italy. Characteristics of enrolled studies are depicted in Table 1. The risk of bias for each included study was assessed as low (Fig. 2 and Figure S1).

**Table 1:** Study characteristics

First author’s name	Year of publication	Country	Number of participants	Age, years	Percentage of the female, %	PSP measurement			Standard reference	
						Method	cutoff		Method	Timepoint at assessment
Llewelyn	2013	UK	162	65.9 (52.0–76)	42	Recombinant human PSP/reg 1α was used to generate an isoform-specific enzyme-linked immunosorbent assay using the sandwich technique [16]	30 ng/ml		The 2001 International Sepsis Definitions Conference definitions of Systemic Inflammatory Response Syndrome (SIRS) and sepsis were used [36], sepsis being defined as SIRS plus either proven infection (on the basis of microbiological sampling or radiology) or probable infection (considering the patient’s clinical presentation, white cell count, CRP, radiology).	NR
Schlapbach	2013	Switzerland	137	0–72 h	37	The levels of PSP were determined using an isoform-specific ELISA [16]	0.1 ng/ml		culture results, perinatal sepsis risk factors, clinical signs of sepsis and laboratory test results [37]	24–72 h after admission
Palmiere	2015	Switzerland	40	54.7 (39–78)	24	The levels of PSP/reg in postmortem serum were determined using a commercialized enzyme-linked immunosorbent assay (ELISA) kit (Homo sapiens REG1a ELISA kit, USCN Life Science Inc., Houston TX, USA) according to manufacturer protocol	1.0 mg/ml		Evidence of pneumonia or abdominal infection along with the presence of systemic inflammatory response syndrome (SIRS) according to the definition by the American College of Chest Physicians/Society of Critical Care Medicine (ACCP/SCCM) [38]	NR
Rass	2016	Egypt	104	12–72 h	NR	PSP which was measured using a double-antibody sandwich enzyme-linked immunosorbent assay (ELISA) kits (MyBiosource/ MBS285689, San Diego, California, USA)	12.96 ng/mL		Perinatal sepsis risk factors, clinical signs of sepsis, results of conventional laboratory tests (WBC < 10,000 or > 26,000/𝜇L), immature leukocyte count > 10%, platelet count < 150,000/𝜇L, CRP (> 5 mg/L), and culture results	24–72 h after admission
García de Guadiana-Romualdo	2017	Spain	152	66 (16–97)	42	PSP levels were measured on microplate assays, using a sandwich isoform-specific enzyme-linked immunoabsorbent assay (ELISA) [16]	96.6 ng/mL		Sepsis-3 criteria [3]	NR
Klein	2021	Switzerland	90	48.5 ± 18.8	20	The concentration of PSP/REG Iα was measured with an isoform specific enzyme-linked immunosorbent assay [39, 40]	60.12 ng/mL		Sepsis-3 criteria [3]	First 10 days after trauma
Pugin	2021	France, Switzerland, Italy, and the UK	243	65.0 (54.0–73.0)	37				The Sepsis-3 criteria were used to define sepsis, and microbiological procedures were performed to diagnose infections responsible for sepsis according to local protocol [3]	
de Hond	2022	Netherlands	156	60.0 (44.5–73.0)	47	PSP values were measured with the CE-marked IVD PSP capsule on abioSCOPE® platform (Abionic SA, Epalinges, Switzerland)	500 ng/mL		Likelihood of infection was assessed using a predefined, four-point scale (ascending from none, possible, probable, to definite), as described by the Centers for Disease Control and International Sepsis Forum Consensus Conference [41]	NR
Saleh	2023	Egypt	280	13	58	PSP values were analyzed using enzyme-linked immunosorbent assay (ELISA) kits (copeptin kit: Shanghai SunRed Biological Technology Co., Shanghai, China; PSP kit: Human PSP, China; APOA5 kit: MultiScience [Lianke] Biotech, Co., Ltd., Hangzhou, China).	9.79 ng/mL		International Pediatric Sepsis Consensus Conference, characterizing septic, severe septic, and septic shock groups [42]	NR

NR, not reported

### Diagnostic performance of PSP

**Fig. 3:**
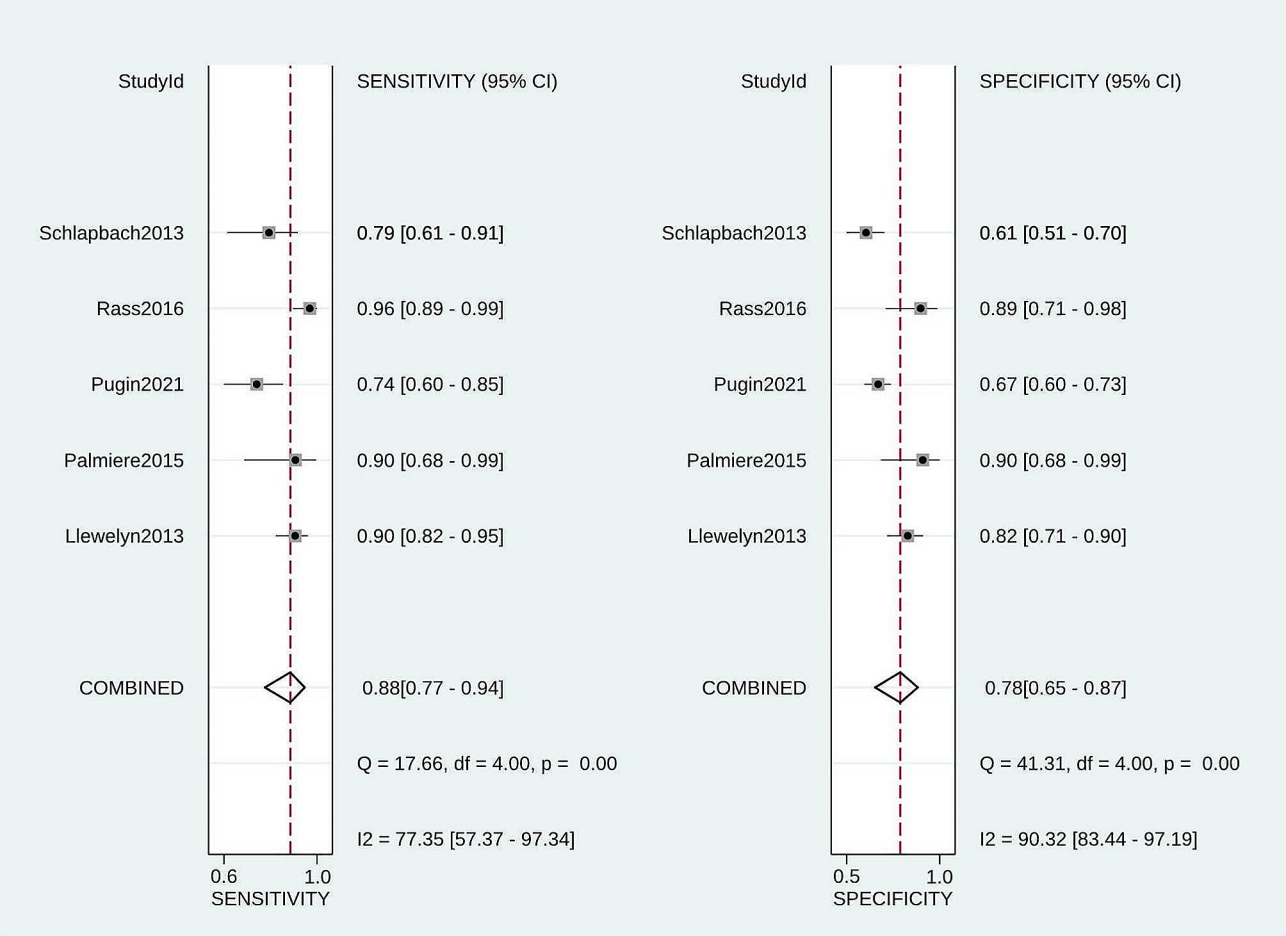
Forest plot of diagnostic performance of PSP in detecting sepsis

**Fig. 4:**
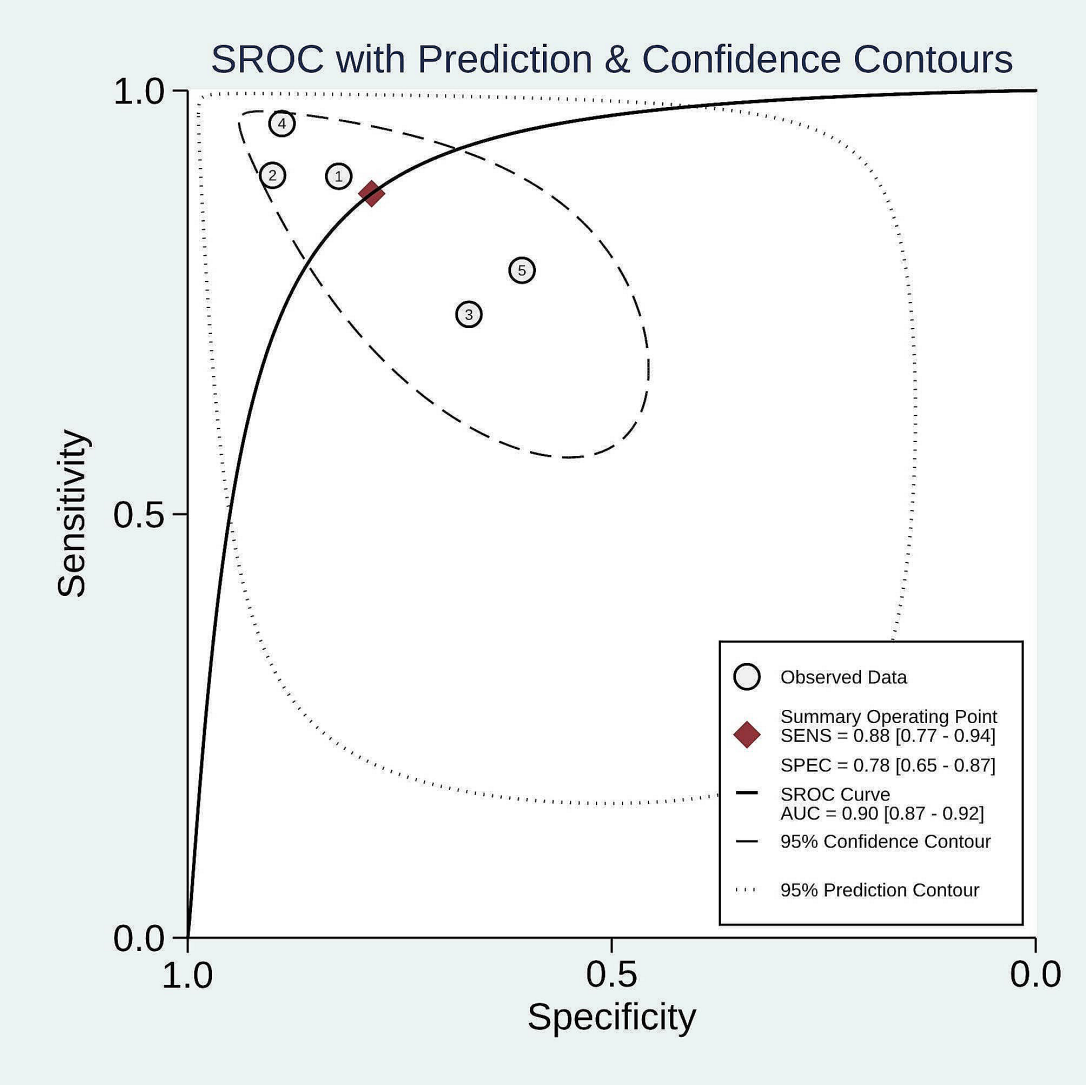
SROC of PSP in detecting sepsis

The overall sensitivity of PSP was 0.88 (95% CI: 0.77–0.94, I^2^ = 77.4%, *p* < 0.01), and the pooled specificity was 0.78 (95% CI: 0.65–0.87, I^2^ = 90.3%, *p* < 0.01), respectively (Fig. 3). Pooled + LR, -LR, and DOR were 4.1 (2.3, 7.3), 0.16 (0.07, 0.34), and 26 (7, 98). The AUC value for the SROC of PSP was 0.90 (0.87, 0.92) (Fig. 4). The pooled sensitivity, specificity, + LR and - LR, and DOR for PSP among neonates were 0.91 (95% CI: 0.84, 0.96), 0.66 (95% CI: 0.58, 0.74), 3.97 (95% CI: 0.53, 29.58), 0.13 (95% CI: 0.02, 1.00), and 31.27 (95% CI: 0.97, 1004.60) (Table 2). The pooled sensitivity, specificity, + LR and - LR, and DOR for PSP among adults were 0.85 (95% CI: 0.78, 0.90), 0.72 (95% CI: 0.67, 0.776), 4.09 (95% CI: 1.69, 9.92), 0.19 (95% CI: 0.08, 0.49), and 22.74 (95% CI: 4.25, 121.73) (Table 2).

**Table 2:** Diagnostic value of PSP in neonates and adults

Population	Number of participants	Sensitivity	Specificity	Positive Likelihood Ratio	Negative Likelihood Ratio	Diagnostic Odds Ratio
Neonates	241	0.91 (0.84, 0.96)	0.66 (0.58, 0.74)	3.97 (0.53, 29.58)	0.13 (0.02, 1.00)	31.27 (0.97, 1004.6)
Adults	445	0.85 (0.78, 0.90)	0.72 (0.67, 0.78)	4.09 (1.69, 9.92)	0.19 (0.08, 0.49)	22.74 (4.25, 121.73)

Data in parentheses were 95% confidence intervals

In the study by Garcia de Guadiana-Romualdo et al., results of the ROC curve analysis revealed an AUC of 0.872 for sepsis identification [11]. Klein et al’s analysis of biomarker kinetics (PSP, routine markers) was performed on 90 burned patients, PSP identified between sepsis, infection and sterile inflammation with an AUC 0.89 [28]. An AUC of 0.69 was reported in de Hond et al’s study including 156 participants [8]. Moreover, in Saleh et al’s study, the ROC revealed that the AUC for PSP reached 0.868 for sepsis diagnosis [29].

### Publication bias

**Fig. 5:**
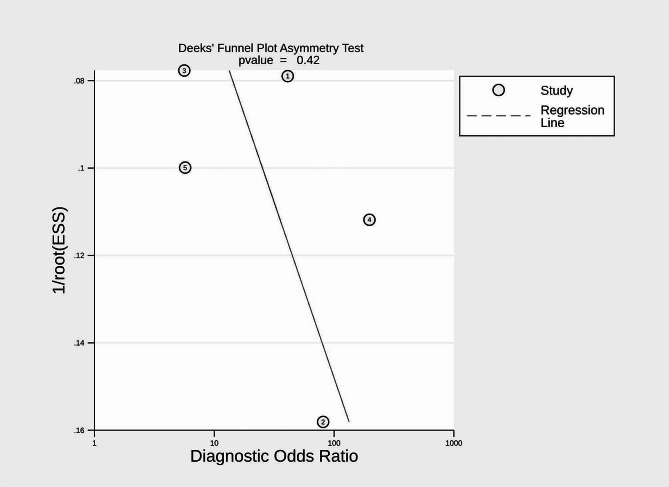
Funnel plot of meta-analysis of PSP in detecting sepsis

Deek’s tests for publication bias yielded *p*-values of 0.42 for the meta-analysis, indicating no statistically significant publication bias (Fig. 5).

### Sensitivity analysis

Results of sensitivity analysis demonstrated that the two included studies investigating the diagnostic value of PSP in neonatal sepsis had a significant impact on the overall effect size, and thus subgroup analysis based on the age of the study population was performed (Figure S2).

## Discussion

Results of this study demonstrated an overall sensitivity of 0.88 (95% CI: 0.77–0.94) and a pooled specificity of 0.78 (95% CI: 0.65–0.87) with an overall AUC value of 0.90 (0.87, 0.92). Compared to other biomarkers, the pooled sensitivity in this meta-analysis was higher than the pooled sensitivity (0.79) in Chen et al’s meta-analysis investigating neutrophil to lymphocyte ratio (NLR) in the diagnosis of sepsis, while the specificity was lower than that (0.91) in Chen et al’s meta-analysis [30]; the comparison between PSP and calprotectin (sensitivity: 0.88 vs. 0.77, specificity: 0.78 vs.0.85) was similar to NLR [31]; Poggi’s meta-analysis assessing the accuracy of presepsin for the sepsis diagnosis showed that the pooled sensitivity and specificity were 0.93 (95% CI, 0.86–0.95) and 0.91 (95% CI, 0.85–0.95), respectively, which were higher than PSP [32]; the overall sensitivity and specificity were higher than those of CRP and PCT [33].

Several research findings suggest that PSP is involved in the early defense mechanism of sepsis [16, 19, 28]. In these studies, PSP has been shown to be associated with the severity of inflammation and can activate neutrophils by upregulating activation markers CD11b and CD62L [16]. In addition to the activation of neutrophils, PSP possesses antibacterial functions; it can induce bacterial aggregation, which may help prevent bacteria from penetrating the intestinal barrier [34]. Moreover, PSP levels rise 72 h before the onset of clinical symptoms of sepsis [8]. The results of this meta-analysis verified that PSP displayed favorable diagnostic performance in detecting sepsis. The results were consistent with previous studies regarding the diagnostic value of PSP in sepsis [8, 11, 29]. Notably, neonatal sepsis is difficult to diagnose due to the nonspecific clinical signs in response to sepsis [35]. Schlapbach et al.‘s study are the first to investigate PSP in neonatal sepsis; results demonstrated that the level of PSP in infected infants was significantly higher than in uninfected ones with an AUC of 0.69 [20]. Subgroup analysis of this meta-analysis showed favorable pooled sensitivity but low specificity for PSP alone in the diagnosis of neonatal sepsis, suggesting the necessity for combining different biomarkers to detect sepsis in this specific population. More well-designed prospective studies are required to clarify this opinion.

Interestingly, the pooled sensitivity for adults was lower than that in neonates, while the difference in specificity is the opposite. The underlying reason needed to be clarified based on the current meta-analysis. However, it was reported that maximum PSP levels in neonates were lower than those in adults with sepsis, which may be one of the reasons for the different diagnostic values for PSP in newborns and adults. Relevant studies are warranted to investigate this difference.

### Strengths and limitations

To our knowledge, this is the first meta-analysis to evaluate the diagnostic value of PSP in the context of sepsis in general. Data from previously published citations were synthesized to enhance the statistical power of the diagnostic value of PSP. Results of this study were favorable and promising, which may serve as both advanced level of evidence and reference for practitioners to make decisions on the diagnosis of sepsis in their clinical practice.

Like other meta-analyses, there are several limitations of this meta-analysis. The study protocol was not registered on PROSPERO. Significant heterogeneity existed between included studies; it may be attributed to differences in the study population, standard reference, and cutoffs of PSP in component studies. Although Deek’s funnel plots asymmetry test revealed no statistically significant publication bias in the meta-analysis, bias caused by published and unpublished studies inherently existed because this study is only focused on published articles. The number of included studies was limited owing to the inclusion criteria of this meta-analysis; aside from subgroup analysis based on the study population, subgroup analysis on other covariates was not performed. The interpretation of findings from this study ought to be with caution; more similar studies are needed to specify the diagnostic value of PSP in detecting sepsis.

## Conclusion

In this meta-analysis, evidence suggests that PSP was a promising biomarker for diagnosing patients suspected of sepsis. According to the findings presented in this meta-analysis, specifically designed studies on different populations are needed to ascertain the validity of PSP measurement to guide early empirical antibiotic treatment, particularly in neonates.

### Electronic supplementary material

Below is the link to the electronic supplementary material.

Supplementary Material 1

Supplementary Material 2

Supplementary Material 3

Supplementary Material 4

## Data Availability

All data generated or analyzed during this study are included in this article and supplementary information files.

## Abbreviations

PSP: pancreatic stone protein
CNKI: China National Knowledge Infrastructure
LR: Likelihood Ratio
DOR: diagnostic odds ratio
AUC: area under the curve
SROC: summary receiver operating characteristic
CRP: C-reactive protein
PCT: Procalcitonin
PRISMA: Preferred Reporting Items for Systematic Reviews and Meta-analysis
TP: true positive
FP: false positive
FN: false negative
TN: true negative
QUADAS-2: Quality Assessment of Diagnostic Accuracy Studies-2
CIs: confidence intervals
