# Supplementary figures and images for: Diagnostic accuracy of pancreatic stone protein in patients with sepsis: a systematic review and meta-analysis
Source: BMC Infect Dis. 2024 May 6;24:472. doi: 10.1186/s12879-024-09347-4 (PMC11071224; doi:10.1186/s12879-024-09347-4)

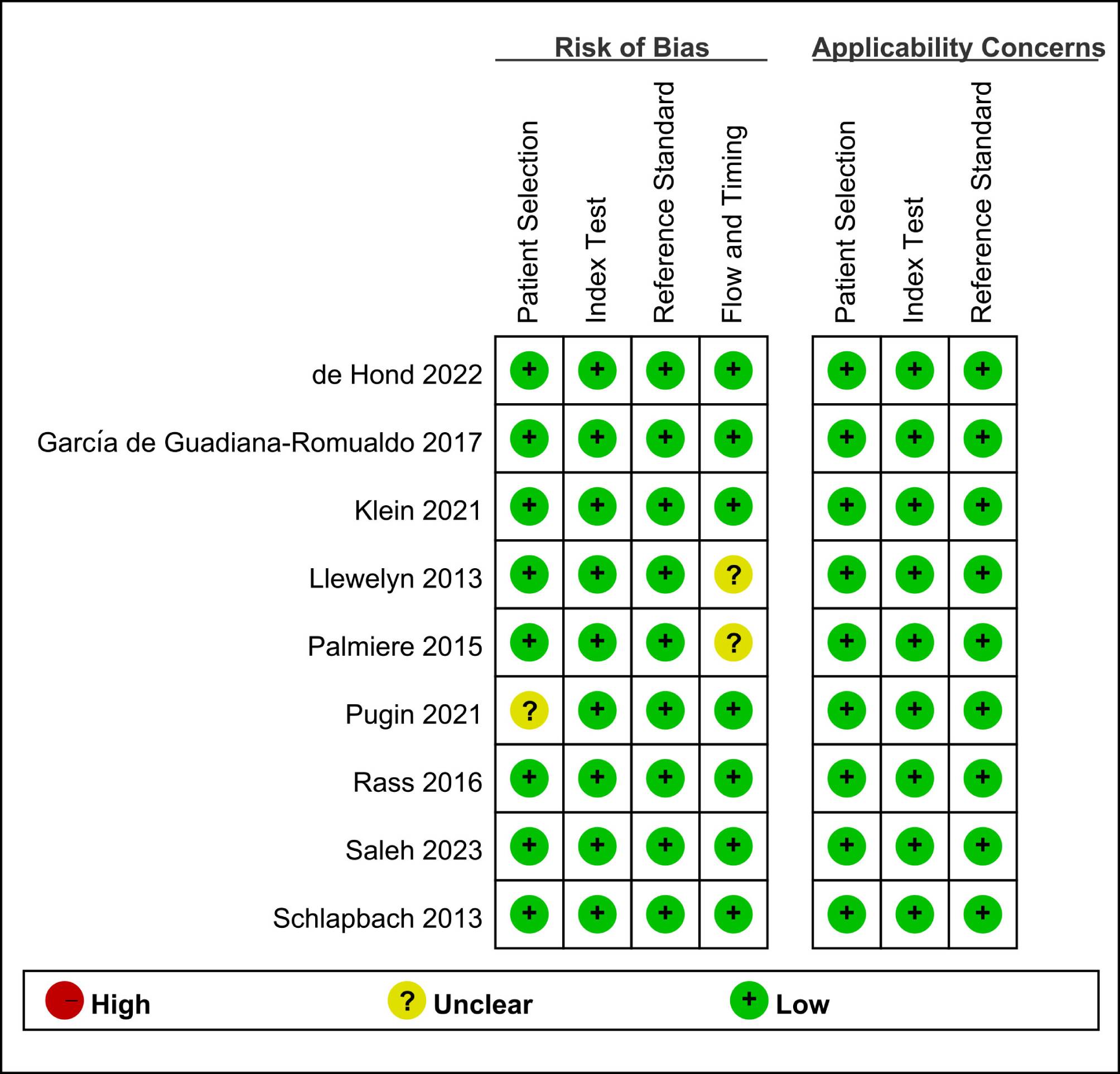

Supplement: Supplementary file 1 — Supplementary Material 1 [file 12879_2024_9347_MOESM1_ESM.jpg]

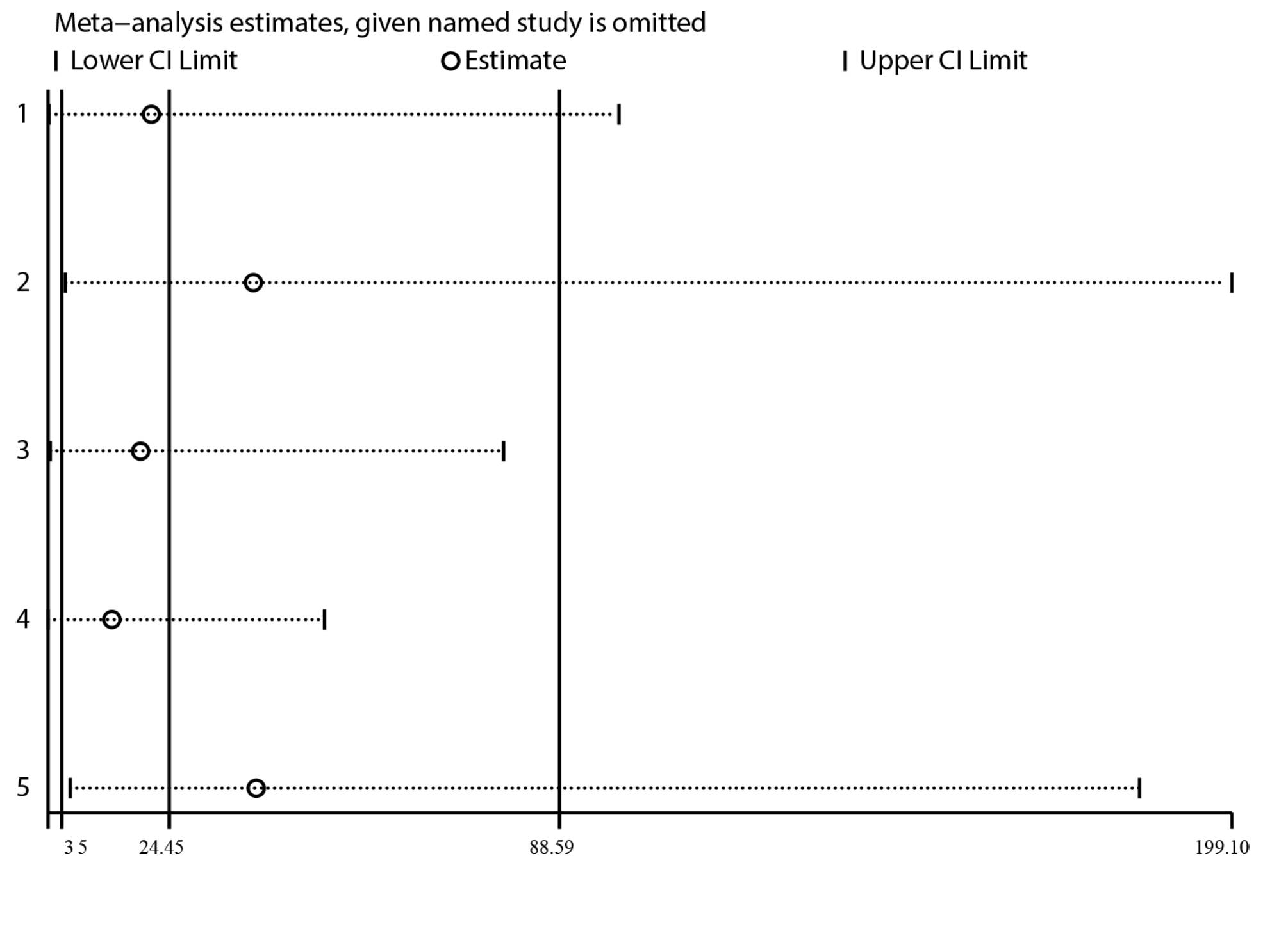

Supplement: Supplementary file 2 — Supplementary Material 2 [file 12879_2024_9347_MOESM2_ESM.jpg]
