# Supplementary material for: Diagnostic accuracy of pancreatic stone protein in patients with sepsis: a systematic review and meta-analysis
Source: BMC Infect Dis. 2024 May 6;24:472. doi: 10.1186/s12879-024-09347-4 (PMC11071224; doi:10.1186/s12879-024-09347-4)
Supplement: Supplementary file 3 — Supplementary Material 3 [file 12879_2024_9347_MOESM3_ESM.docx]

**Figure S1.** Risk of bias and applicability concerns summary for each included study.

**Figure S2.** Results of sensitivity analysis
