# Supplementary material for: Diagnostic accuracy of pancreatic stone protein in patients with sepsis: a systematic review and meta-analysis
Source: BMC Infect Dis. 2024 May 6;24:472. doi: 10.1186/s12879-024-09347-4 (PMC11071224; doi:10.1186/s12879-024-09347-4)
Supplement: Supplementary file 4 — Supplementary Material 4 [file 12879_2024_9347_MOESM4_ESM.docx]

**Table S1. Search strategies for databases**

**Search strategy of PubMed**

| Search number | Query | Sort By | Filters | Search Details | Results | Time |
| --- | --- | --- | --- | --- | --- | --- |
| 3 | ((pancreatic stone protein[Title/Abstract]) OR (PSP[Title/Abstract]) AND (english[Filter])) AND (((((((((((((((((((sepsis[MeSH Terms]) OR (sepsis[Title/Abstract])) OR (Bloodstream Infection[Title/Abstract])) OR (Bloodstream Infections[Title/Abstract])) OR (Infection, Bloodstream[Title/Abstract])) OR (Pyemia[Title/Abstract])) OR (Pyemias[Title/Abstract])) OR (Pyohemia[Title/Abstract])) OR (Pyohemias[Title/Abstract])) OR (Pyaemia[Title/Abstract])) OR (Pyaemias[Title/Abstract])) OR (Septicemia[Title/Abstract])) OR (Septicemias[Title/Abstract])) OR (Poisoning, Blood[Title/Abstract])) OR (Blood Poisoning[Title/Abstract])) OR (Blood Poisonings[Title/Abstract])) OR (Poisonings, Blood[Title/Abstract])) OR (Severe Sepsis[Title/Abstract])) OR (Sepsis, Severe[Title/Abstract]) AND (english[Filter])) | | English | (("pancreatic stone protein"[Title/Abstract] OR "PSP"[Title/Abstract]) AND "english"[Language] AND (("Sepsis"[MeSH Terms] OR "Sepsis"[Title/Abstract] OR "bloodstream infection"[Title/Abstract] OR "bloodstream infections"[Title/Abstract] OR "infection bloodstream"[Title/Abstract] OR "Pyemia"[Title/Abstract] OR "Pyemias"[Title/Abstract] OR "Pyohemia"[Title/Abstract] OR "Pyohemias"[Title/Abstract] OR "Pyaemia"[Title/Abstract] OR "Septicemia"[Title/Abstract] OR "Septicemias"[Title/Abstract] OR "poisoning blood"[Title/Abstract] OR "blood poisoning"[Title/Abstract] OR "blood poisonings"[Title/Abstract] OR (("poisoned"[All Fields] OR "Poisoning"[MeSH Terms] OR "Poisoning"[All Fields] OR "Poisonings"[All Fields] OR "Poisoning"[MeSH Subheading] OR "poisonous"[All Fields] OR "poisons"[Pharmacological Action] OR "poisons"[MeSH Terms] OR "poisons"[All Fields] OR "poison"[All Fields]) AND "Blood"[Title/Abstract]) OR "severe sepsis"[Title/Abstract] OR "sepsis severe"[Title/Abstract]) AND "english"[Language])) AND (english[Filter]) | 71 | 21:42:39 |
| 2 | ((((((((((((((((((sepsis[MeSH Terms]) OR (sepsis[Title/Abstract])) OR (Bloodstream Infection[Title/Abstract])) OR (Bloodstream Infections[Title/Abstract])) OR (Infection, Bloodstream[Title/Abstract])) OR (Pyemia[Title/Abstract])) OR (Pyemias[Title/Abstract])) OR (Pyohemia[Title/Abstract])) OR (Pyohemias[Title/Abstract])) OR (Pyaemia[Title/Abstract])) OR (Pyaemias[Title/Abstract])) OR (Septicemia[Title/Abstract])) OR (Septicemias[Title/Abstract])) OR (Poisoning, Blood[Title/Abstract])) OR (Blood Poisoning[Title/Abstract])) OR (Blood Poisonings[Title/Abstract])) OR (Poisonings, Blood[Title/Abstract])) OR (Severe Sepsis[Title/Abstract])) OR (Sepsis, Severe[Title/Abstract]) | | English | ("Sepsis"[MeSH Terms] OR "Sepsis"[Title/Abstract] OR "bloodstream infection"[Title/Abstract] OR "bloodstream infections"[Title/Abstract] OR "infection bloodstream"[Title/Abstract] OR "Pyemia"[Title/Abstract] OR "Pyemias"[Title/Abstract] OR "Pyohemia"[Title/Abstract] OR "Pyohemias"[Title/Abstract] OR "Pyaemia"[Title/Abstract] OR "Septicemia"[Title/Abstract] OR "Septicemias"[Title/Abstract] OR "poisoning blood"[Title/Abstract] OR "blood poisoning"[Title/Abstract] OR "blood poisonings"[Title/Abstract] OR (("poisoned"[All Fields] OR "Poisoning"[MeSH Terms] OR "Poisoning"[All Fields] OR "Poisonings"[All Fields] OR "Poisoning"[MeSH Subheading] OR "poisonous"[All Fields] OR "poisons"[Pharmacological Action] OR "poisons"[MeSH Terms] OR "poisons"[All Fields] OR "poison"[All Fields]) AND "Blood"[Title/Abstract]) OR "severe sepsis"[Title/Abstract] OR "sepsis severe"[Title/Abstract]) AND (english[Filter]) | 220,043 | 21:41:54 |
| 1 | (pancreatic stone protein[Title/Abstract]) OR (PSP[Title/Abstract]) | | English | ("pancreatic stone protein"[Title/Abstract] OR "PSP"[Title/Abstract]) AND (english[Filter]) | 7,264 | 21:39:06 |

**Search strategy of EMBASE**

| No. | Query | Results | Date |
| --- | --- | --- | --- |
| #4 | #1 AND #2 AND [english]/lim | 143 | 20-Apr-23 |
| #3 | #1 AND #2 | 153 | 20-Apr-23 |
| #2 | sepsis:ab,ti OR 'sepsis'/exp OR septicemia:ab,ti OR 'septic shock':ab,ti | 382547 | 20-Apr-23 |
| #1 | 'pancreatic stone protein'/exp OR 'pancreatic stone protein' OR (pancreatic AND ('stone'/exp OR stone) AND ('protein'/exp OR protein)) OR psp:ab,ti | 12237 | 20-Apr-23 |

**Search strategy of Cochrane Library**

Date Run: 20/04/2023 10:18:54

ID Search Hits

#1 (pancreatic stone protein):ti,ab,kw OR (PSP):ti,ab,kw (Word variations have been searched) 732

#2 MeSH descriptor: [Sepsis] explode all trees 6710

#3 (sepsis):ti,ab,kw OR (Septicemia):ti,ab,kw (Word variations have been searched) 13739

#4 #2 OR #3 16402

#5 #1 AND #4 2

**Search strategy of Web of Science**

| # | Search Query | Database | Results | Date Run |
| --- | --- | --- | --- | --- |
| 1 | pancreatic stone protein (Topic) | Web of Science Core Collection | 433 | Mon Apr 20 2023 10:08:39 GMT+0800 (中国标准时间) |
| 2 | sepsis (Topic) OR Septicemia (Topic) | Web of Science Core Collection | 162668 | Mon Apr 20 2023 10:08:39 GMT+0800 (中国标准时间) |
| 3 | #1 AND #2 | Web of Science Core Collection | 59 | Mon Apr 20 2023 10:08:39 GMT+0800 (中国标准时间) |
| 4 | #1 AND #2 and Article (Document Types) | Web of Science Core Collection | 42 | Mon Apr 20 2023 10:08:39 GMT+0800 (中国标准时间) |
| 5 | #1 AND #2 and Article (Document Types) and English (Languages) | Web of Science Core Collection | 39 | Mon Apr 20 2023 10:08:39 GMT+0800 (中国标准时间) |
